# Transcriptional analysis of arogenate dehydratase genes identifies a link between phenylalanine biosynthesis and lignin biosynthesis

**DOI:** 10.1093/jxb/eraa099

**Published:** 2020-02-24

**Authors:** Jorge El-Azaz, Fernando de la Torre, María Belén Pascual, Sandrine Debille, Francis Canlet, Luc Harvengt, Jean-François Trontin, Concepción Ávila, Francisco M Cánovas

**Affiliations:** 1 Grupo de Biología Molecular y Biotecnología de Plantas (BIO-114), Universidad de Málaga, Málaga, Spain; 2 Institut Technologique FCBA, Pôle Biotechnologies et Sylviculture Avancée (BSA), Pierroton, Cestas, France; 3 Simon Turner, University of Manchester, UK

**Keywords:** Amino acids, phenylalanine metabolism, phenylpropanoids, *Pinus pinaster*, transcription factors, transgenic trees

## Abstract

Biogenesis of the secondary cell wall in trees involves the massive biosynthesis of the phenylalanine-derived polymer lignin. Arogenate dehydratase (ADT) catalyzes the last, and rate-limiting, step of the main pathway for phenylalanine biosynthesis. In this study, we found that transcript levels for several members of the large ADT gene family, including *ADT-A* and *ADT-D*, were enhanced in compression wood of maritime pine, a xylem tissue enriched in lignin. Transcriptomic analysis of maritime pine silenced for *PpMYB8* revealed that this gene plays a critical role in coordinating the deposition of lignin with the biosynthesis of phenylalanine. Specifically, it was found that *ADT-A* and *ADT-D* were strongly down-regulated in PpMYB8-silenced plants and that they were transcriptionally regulated through direct interaction of this transcription factor with regulatory elements present in their promoters. Another transcription factor, PpHY5, exhibited an expression profile opposite to that of PpMYB8 and also interacted with specific regulatory elements of *ADT-A* and *ADT-D* genes, suggesting that it is involved in transcriptional regulation of phenylalanine biosynthesis. Taken together, our results reveal that PpMYB8 and PpHY5 are involved in the control of phenylalanine formation and its metabolic channeling for lignin biosynthesis and deposition during wood formation in maritime pine.

## Teaser

Functional genomics unravels the role of two arogenate dehydratases in maritime pine during wood formation, and provides new insights into how lignin biosynthesis and deposition are regulated in conifers.

## Introduction

The biosynthesis of the amino acid phenylalanine (Phe) is a doubly essential process for land plants, as this amino acid serves both as a building block for proteins and as a main precursor for the biosynthesis of phenylpropanoids. Phenylpropanoids are a wide range of aromatic compounds that play key roles in plant growth, development, and the response to environmental cues. Land colonization by the first terrestrial plants would not have been possible without the emergence of specialized metabolic pathways for the biosynthesis of these secondary metabolites (Lowry *et al.*, 1980). The metabolism of Phe is critical in carbon channeling from photosynthesis to the biosynthesis of phenylpropanoids in conifers, mainly lignin, which is an important constituent of wood (Pascual *et al.*, 2016). A tight regulation of Phe metabolic flux would be expected depending on its alternative use for protein biosynthesis versus phenylpropanoid biosynthesis. In vascular plants, this second fate involves a massive carbon flux with as much as 30% of photosynthetically fixed carbon towards the synthesis of lignin and other compounds (Haslam, 1993).

**Fig. 1.**
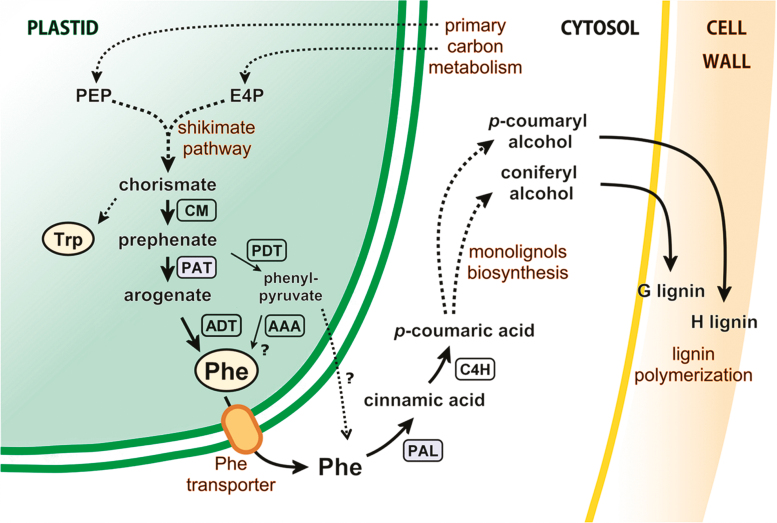
Pathways for aromatic amino acid and lignin biosynthesis in conifers. Enzyme abbreviations are indicated within rectangles. Blue rectangles indicate enzymes encoded by genes previously demonstrated to be regulated by PpMYB8 (Craven-Bartle *et al.*, 2013). PEP, phosphoenolpyruvate; E4P, erythrose 4-phosphate; CM, chorismate mutase; PAT, prephenate aminotransferase; PDT, prephenate dehydratase; ADT, arogenate dehydratase; ADH, arogenate dehydrogenase; AAA, aromatic amino acid aminotransferase; PAL, phenylalanine ammonia-lyase; C4H, cinnamic acid 4-hydroxylase. Phenylpyruvate transport outside the plastid needs to be confirmed.

Within the plastids, primary carbon metabolism is connected to the biosynthesis of aromatic amino acids via the shikimate pathway (Fig. 1). This pathway starts with phospho*enol*pyruvate (PEP) and erythrose-4-phosphate (E4P), which are ultimately converted into chorismate, the direct precursor for the biosynthesis of aromatic amino acids and others compounds, such as vitamins K1 and B9 (Fig. 1; Tzin and Galili, 2010). Chorismate is alternatively used by anthranilate synthase in the initial reaction of the tryptophan biosynthetic pathway or by chorismate mutase (CM) to generate prephenate for the biosynthesis of Phe and tyrosine (Cotton and Gibson, 1965; Maeda and Dudareva, 2012). The biosynthesis of Phe occurs by transamination of prephenate to arogenate by the enzyme prephenate aminotransferase (PAT) and, in a second step, decarboxylation and dehydratation by the enzyme arogenate dehydratase (ADT) to produce Phe (Bonner and Jensen, 1987). Alternatively, Phe can also be synthesized through the decarboxylation and dehydration of prephenate into phenylpyruvate by the enzyme prephenate dehydratase (PDT), which is then followed by the conversion of phenylpyruvate into Phe by an aromatic amino acid aminotransferase (AAA) (Fischer and Jensen, 1987; Fig. 1). While in most microorganisms and fungi Phe biosynthesis occurs through the phenylpyruvate-dependent pathway, the arogenate pathway has been proposed as being predominant in plants (Maeda et al., 2010, 2011) (Fig. 1). Later, Phe is channeled into phenylpropanoid metabolism by the commitment reaction catalyzed by phenylalanine ammonia-lyase (PAL), producing cinnamic acid (Mottiar *et al.*, 2016). Finally, the monolignols *p*-coumaroyl alcohol and coniferyl alcohol will constitute the building blocks for biosynthesis of H-lignins and G-lignins, respectively (Fig. 1). Sinapyl alcohol, the S-lignin monomer, is absent in conifers due to the lack of feruloyl-5-hydroxylase (F5H) activity in this group of plants (Wagner *et al.*, 2015; Pascual *et al.*, 2016).

The regulation of biosynthesis of phenylpropanoids and lignin in plants is a complex process that involves the coordinated expression of genes encoding enzymes located in different subcellular compartments and cellular types. Vascular plants, from herbaceous species to trees, share a relatively conserved ancestral xylem transcriptome (Li *et al.*, 2010). Thus, our current knowledge highlights a general pyramid-shaped regulatory scenario in which a limited set of transcription factors (TFs), placed at the top of the regulatory network, act as master regulators of a larger set of downstream TFs with more specific regulatory functions over particular cell wall biosynthetic genes (Nakano *et al.*, 2015). This hierarchical model is based on two extensively characterized TF families: NACs and MYBs (Zhong *et al.*, 2011). In this model, NAC TFs act as master regulators, controlling the expression of MYB TFs, one of the largest TF families found in plants (Martin and Paz-Ares, 1997). In this regard, we have recently reported that the TF, PpNAC1, acts as a main regulator of Phe biosynthesis and utilization in maritime pine (Pascual *et al.*, 2018).

Members of the MYB family regulate lignification through interactions with AC elements present in the promoter regions of phenylpropanoid and lignin biosynthetic genes (Zhong and Ye, 2009). In trees, several studies have demonstrated the roles of various TFs of this family in wood formation. For example, EgMYB88 and EgMYB1 have been reported to control lignin biosynthesis in eucalyptus (Soler et al., 2016, 2017). In conifers, PtMYB1, PtMYB4, and PtMYB8 have been shown to regulate phenylpropanoid metabolism and secondary cell wall biogenesis in *Pinus taeda* by controlling multiple genes encoding phenylpropanoid enzymes involved in lignin monomer synthesis (Patzlaff et al., 2003*a*, *b*; Bomal *et al.*, 2008), similar to PgMYB1 and PgMYB8 in *Picea glauca* (Bomal *et al.*, 2014). In addition, Craven-Bartle *et al.* (2013) reported the capacity of PpMYB8 from *P. pinaster* to coactivate the expression of *PpPAT* and *PpPAL* by specific binding to a conserved AC-II element in the promoter region of these genes. *PpMYB8* transcripts were also abundant in lignifying stem tissues, suggesting the existence of a conserved transcriptional network controlling such processes in conifers.

Despite the fact that they can act as a bottleneck in Phe biosynthesis (Maeda and Dudareva, 2012; Corea *et al.*, 2012), and therefore as a constraining factor in wood formation, little attention has been paid to the regulation of genes involved in Phe biosynthesis in trees. In the present work, we have characterized two *ADT* genes in maritime pine, *PpADT-A* and *PpADT-D*, which are highly expressed during formation of compression wood (CW), a specialized vascular tissue with an enhanced deposition of lignin (Villalobos *et al.*, 2012). The analysis of transgenic pine silenced for *PpMYB8*, along with TF–promoter interaction experiments, revealed that PpMYB8 coordinates the expression of *PpADT-A* and *PpADT-D* with lignification, supporting the hypothesis that both ADT isoforms have a relevant role in the biosynthesis of Phe that is then channeled into lignin. Based on these studies, we have found that PpHY5, an ortholog of Arabidopsis AtHY5 (At5g11260) that belongs to the bZIP family of TFs, is also able to bind to the regulatory regions of the *PpADT-A* and *PpADT-D* genes. Interestingly, the gene expression pattern of *PpHY5* is opposite to that of *PpMYB8* in CW, suggesting an opposite role for these two genes in lignification.

## Materials and methods

### Generation of *Pinus pinaster PpMYB8* transgenic lines

All *P. pinaster* transgenic plants were derived from the PN519 embryogenic line (Breton *et al.*, 2006; Trontin *et al.*, 2016). For the overexpression (OE) of *PpMYB8*, the corresponding cDNA was integrated into the pMBb7Fm21GW-UBIL vector (Karimi *et al.*, 2002) and for the *PpMYB8* RNAi-mediated silencing, a 243 bp fragment from the 3' end of *P. taeda MYB8* was subcloned into the pB7GWIWG2(II) vector. Transformation was performed by co-cultivation of embryonal suspensor masses with *Agrobacterium tumefaciens* carrying the corresponding OE or RNAi vectors (Trontin *et al.*, 2002). Transgenic lines were selected with phosphinothricin (PPT), confirmed by PCR assays (Trontin et al., 2007, 2013), and cryopreserved (Harvengt, 2005). Somatic embryo development from selected cryopreserved transgenic embryogenic lines was achieved on mLV-based maturation medium within 12 weeks following the method reported in Morel *et al.* (2014). A detailed description of methods used for maritime pine transformation and regeneration is provided in Supplementary Protocols S1 at *JXB* online.

### Cloning of gene regulatory regions

*PpADT-A* and *PpADT-D* upstream regulatory sequences were obtained from SustainPineDB and cloned using 100 ng of genomic DNA of *P. pinaster* seedlings as template. A nested-PCR approach was used to amplify the *ADT-A* upstream region, using the primer pairs (see Supplementary Table S2): p1830.5Fwd/p1830.7Rvs (first PCR) and p1830.4Fwd/p1830.6Rvs (second PCR). The *ADT-D* upstream region was amplified in a single PCR by using the primer pair p3030.2Fwd/p3030Rvs. PCR products were purified from the gel, cloned into pJET1.2/blunt (Thermo Fisher Scientific), and confirmed by sequencing.

### Reverse transcription–quantitative PCR (RT–qPCR) analysis

Total RNA was isolated from maritime pine tissues and cDNA was synthesized as described previously (de la Torre *et al.*, 2014). Samples of compression (CW) and opposite (OW) wood were collected from 25-year-old pine trees growing in Sierra Bermeja (Estepona, Spain) and sampled in May 2008 (Villalobos *et al.*, 2012). Bark and phloem were removed, and developing xylem was carefully scraped with a scalpel. Xylem scrapings were immediately frozen in liquid nitrogen after harvesting and stored at –80 °C. At least three biological replicates were used for transcript quantification. qPCR was performed on a CFX-384 Real Time System (Bio-Rad) with SsoFast EvaGreen Supermix (Bio-Rad) under the following conditions: 95 °C for 2 min (one cycle), followed by 95 °C for 1 s and 60 °C for 5 s (45 cycles). cDNAs corresponding to 10 ng of reverse-transcribed RNA were used as template. Raw fluorescence data from each well were fitted to the Mass Action Kinetic 2 model, which requires no assumptions about the amplification efficiency of a qPCR assay (Boggy and Woolf, 2010). The initial target concentration (*D*_0_ parameter) for each gene was deduced from the Mass Action Kinetic 2 model using the qPCR package for the R environment (Ritz and Spiess, 2008), and normalized to *PpActin2* and *PpEF1α*. All primers used for RT–qPCR are listed in Supplementary Table S3.

### Microarray hybridization and data analysis

The 60-mer oligonucleotides custom microarray PINARRAY3 (Cañas *et al.*, 2015) based on the *P. pinaster* transcriptome (Canales *et al*., 2015) was used. Slides were made by Agilent Technologies and hybridization was performed as described by Cañas *et al.* (2015). Slides were scanned, and signal intensities were recorded using a GenePix 4100A microarray scanner (Molecular Devices, Sunnyvale, CA, USA). Differentially expressed genes (DEGs) were detected using the limma package for R (Smyth, 2005). Gene enrichment comparison was performed using the Mapman functional categories through the Mercator web tool (Lohse *et al.*, 2014). The microarray data are accessible at NCBI’s Gene Expression Omnibus (Edgar *et al.*, 2002) through the accession number GSE142093.

### EMSA

The recombinant protein PpMYB8 (FN868598) was produced in *Escherichia coli* BL21-AI™ (Thermo Fisher Scientific) by overnight culture at 12 °C. The oligonucleotide probes described in Fig. 5 containing the AC elements, and those described in Fig. 7 containing ACGT elements were generated by annealing complementary biotinylated oligonucleotides designed to create 5′-biotinylated amplicons. At the end of the incubation period, the DNA–protein complexes were analysed by electrophoresis as previously described (Rueda-López *et al.*, 2008). The binding specificity was evaluated using competition experiments with the corresponding non-biotinylated DNA probes.

### Plasmid constructions for transcriptional analysis in yeast

Deletions from the 5' end of *PpADT-A* were amplified from the pJET1.2 construct using the reverse primer PAAttB1R paired with three different forward primers: PA1AttB4 (638), PA2AttB4 (394), and PA3AttB4 (246). The corresponding PCR products were re-amplified using the primers AttB4 and AttB1R, and cloned into Gateway^®^ pDONR-P4-P1R (Thermo Fisher Scientific) vector using BP Clonase^®^ II, and subsequently recombined into pMW#3 (Deplancke *et al.*, 2006) using the Gateway^®^ LR Clonase^®^ II mix. Deletions from the 5' end of *PpADT-D* were amplified using the primers: PD1AttB4, PD2AttB4, PD3AttB4 (forward), and PDAttB1R (reverse). *PpADT-D* promoter deletions were subcloned as described for *PpADT-A*. All primers used for promoter analysis in yeast are listed in Supplementary Table S2.

The *PpMYB8* prey construct was done by amplifying the *PpMYB8* ORF from a construct previously available in our laboratory (Craven-Bartle *et al.*, 2013) using the primers MYB8AttB1 and MYB8AttB2. The PCR product was re-amplified using the primers AttB1 and AttB2, cloned into Gateway^®^ pDONR207, and recombined into Gateway^®^ pDEST22 as described.

### Yeast manipulation and β-galactosidase assay

*In vivo* interaction between PpMYB8 and the regulatory regions of *P*_*ADT-A*_ and *P*_*ADT-D*_ was analyzed through a β-galactosidase reporter assay performed in *Saccharomyces cerevisiae* using *o*-nitrophenyl-galactopyranoside (ONPG) as substrate. More details can be found in Supplementary Protocols S1.

### Microscopy

Stems previously preserved in liquid nitrogen were fixed overnight in a 4% paraformaldehyde solution in phosphate-buffered saline (PBS) and included in paraffin. Transversal sections (10 µm) were deparaffinized and rehydrated before visualization. For Wiesner (phloroglucinol-HCl) staining, a few drops of phloroglucinol-HCl solution (two parts of 1% phloroglucinol in 95% ethanol and one part of pure HCl) were deposited over the tissue sections and observed immediately under white light. For lignin autofluorescence visualization, an excitation range of 460–500 nm and an emission range of >510 nm were used. In both cases (Wiesner staining and lignin autofluorescence), a Leica TL3000 Ergo stereo-microscope was used to visualize and register the results.

### Lignin determination

Acid-soluble and -insoluble lignin were determined in cell wall preparations from ~500 mg of fresh ground stems preserved at –80 °C. Acid-insoluble lignin was determined from ~100 mg of cell wall preparation using the Klason method (Dence, 1992) with the modifications described by Saleme *et al.* (2017). Acid-soluble lignin was determined spectrophotometrically as described (Dence, 1992).

## Results

### *PpADT* genes are up-regulated in compression wood

**Fig. 2.**
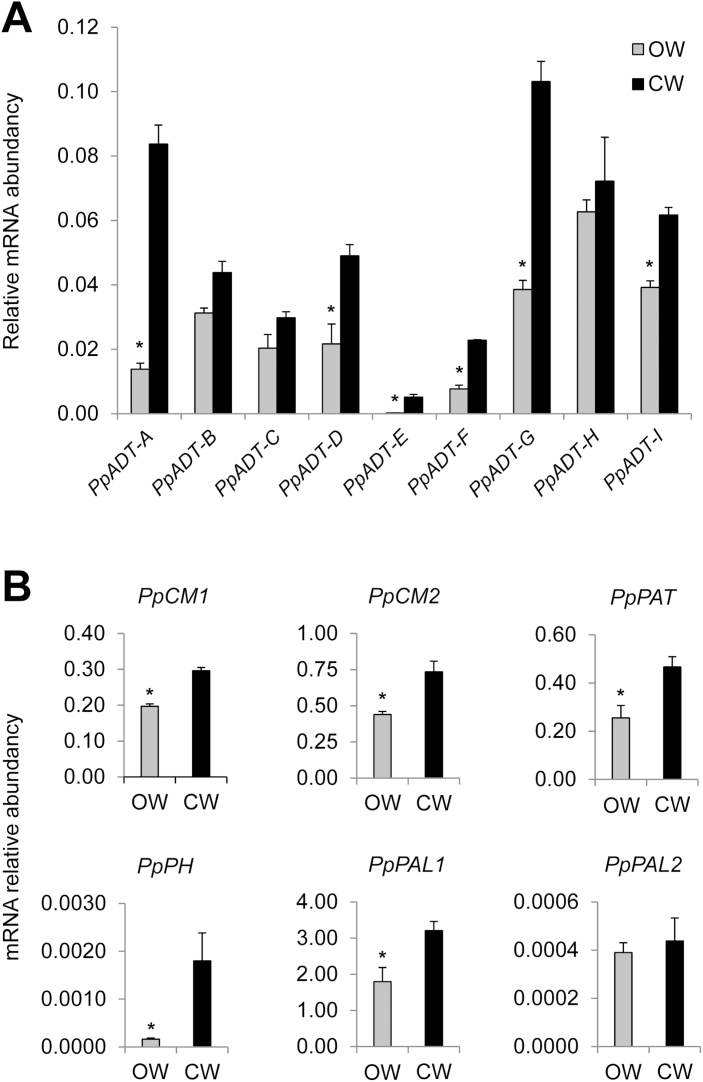
Genes encoding enzymes from Phe metabolism and phenylpropanoid biosynthesis are induced in compression wood (CW) from *Pinus pinaster*. (A) *PpADT* gene relative mRNA levels in opposite wood (OW; gray bars) compared with CW (black bars). (B) Comparison of the expression level of some key Phe and phenylpropanoid biosynthetic genes between OW and CW. *PpCM1*, chorismate mutase 1; *PpCM2*, chorismate mutase 2; *PpPAT*, prephenate aminotransferase; *PpPH*, phenylalanine hydroxylase; *PpPAL1*, phenylalanine ammonia-lyase 1; *PpPAL2*, phenylalanine ammonia-lyase 2. Error bars represent the SD; asterisks indicate significant differences by *t*-test between OW and CW (α*=*0.01; *n*=3).

Conifers develop a specific woody tissue known as CW on the underside of branches and leaning stems. In parallel, facing the formation of the CW, these branches and stems develop OW (Groover, 2016). Compared with OW, in CW the lignin biosynthesis and deposition is enhanced and the cellulose content reduced, representing a good model to study the transcriptional regulation of the metabolic pathways that provide substrates for xylogenesis. In a previous study, we reported the characterization of the *P. pinaster* ADT gene family, consisting of nine identified isoforms (El-Azaz *et al.*, 2016). To determine the *PpADT* isoforms that could be involved in xylogenesis, we have studied the expression profile of the *ADT* gene family in samples of CW and OW that were collected from 25-year-old trees mechanically stressed to produce such specialized vascular tissues (Villalobos *et al.*, 2012). Six of the nine *ADT* genes showed significantly higher expression in CW when compared with OW: *PpADT-A*, *PpADT-D*, *PpADT-E*, *PpADT-F*, *PpADT-G*, and *PpADT-I*. Among them, *PpADT-A*, *PpADT-D*, and *PpADT-G* exhibited the greatest change (Fig. 2A). In CW, enhanced expression levels were also found for genes that encode other enzymes directly involved in the biosynthesis and utilization of Phe and monolignols such as chorismate mutases 1 and 2 (*PpCM1* and *PpCM2*), prephenate aminotransferase (*PpPAT*), phenylalanine hydroxylase (*PpPH*), and phenylalanine ammonia lyase *PpPAL1* (Fig. 2B).

### Production of transgenic lines with altered *PpMYB8* expression in maritime pine

**Fig. 3.**
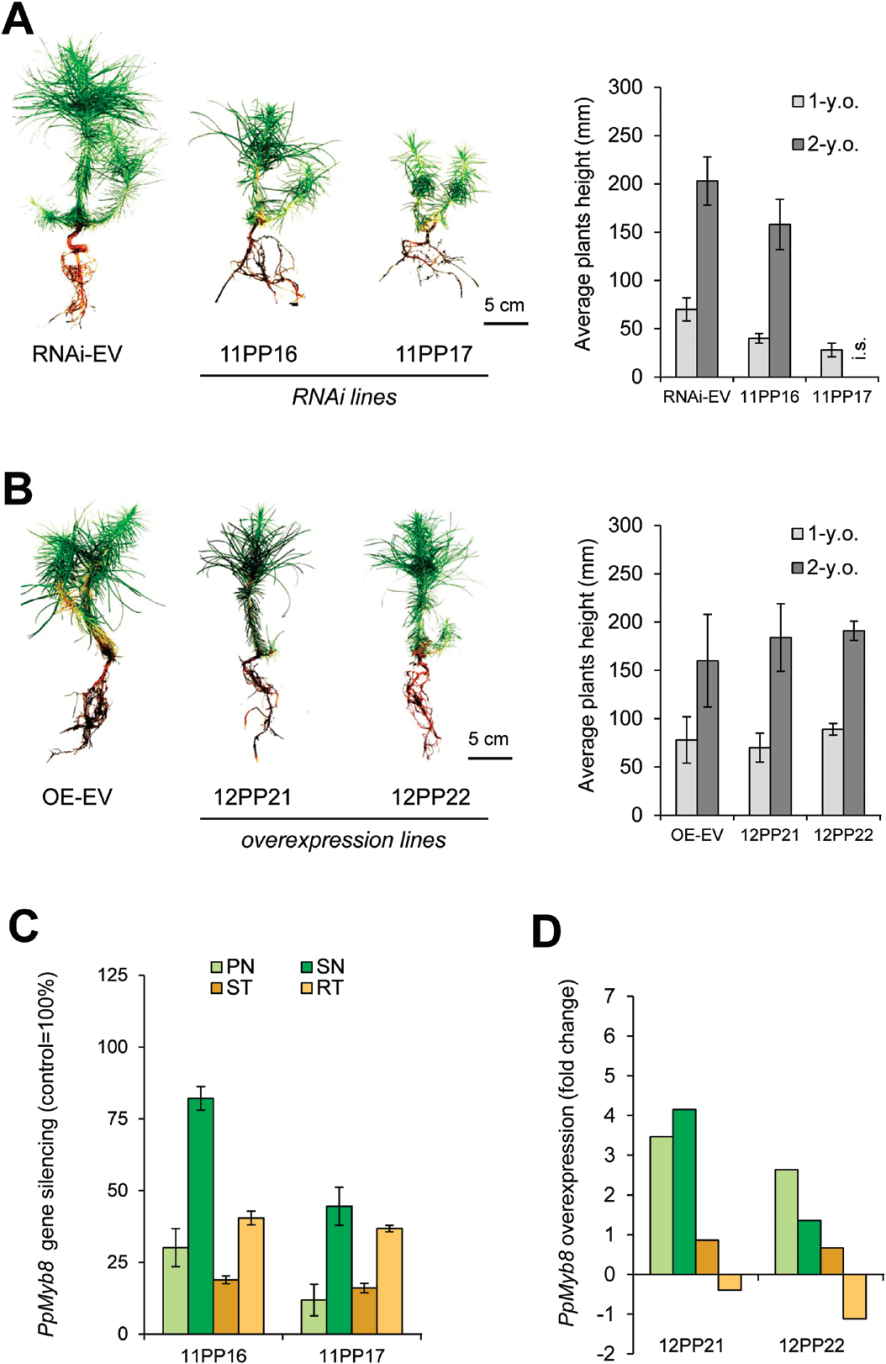
Characterization of transgenic *Pinus pinaster PpMYB8* lines. (A) Morphology after 1 year in the greenhouse, and average height (left), of the transgenic lines (11PP16 and 11PP17) silenced for *PpMYB8* (RNAi-*PpMYB8*). (B) Phenotype of *PpMYB8* overexpression transgenic lines 12PP21 and 12PP22 (OE-*PpMYB8*). (C) Determination of the silencing level of *PpMYB8* in different organs of 12-month-old RNAi-*PpMYB8* plants, expressed as a percentage relative to the expression level of *PpMYB8* in the transgenic control plants carrying the empty silencing vector (RNAi-EV). PN, primary needles; SN, secondary needles; ST, stems; RT, roots. (D) Expression level of *PpMYB8* in the OE-*PpMYB8* lines, as fold change relative to control plants transformed with the empty overexpression vector (OE-EV). Error bars represent the SD; asterisks indicate significant differences by *t*-test between the transgenic line and reference control line (α*=*0.01; *n*=3); i.s., inadequate too low sampling for 11PP17 (data excluded from the statistical analysis).

PpMYB8 has been suggested as a positive regulator of the expression of genes involved in Phe metabolism and nitrogen recycling during xylogenesis in CW (Craven-Bartle *et al.*, 2013). To further explore processes controlled by this TF and identify members of the *ADT* gene family that could be involved in xylogenesis, transgenic plants of maritime pine overexpressing or silenced for *PpMYB8* were obtained from cryopreserved transgenic lines via somatic embryogenesis (Fig. 3). Samples from primary and secondary needles, stems, and roots were collected twice after ~12 and 24 months of growth.

Compared with controls (RNAi-EV), RNAi-*MYB8* plants exhibited a significant decrease in height after 1 year of growth, and differences were still detected after 2 years (Fig. 3A). In contrast, transgenic plants overexpressing OE-*PpMYB8* did not exhibit differences in growth after either 1 or 2 years in the greenhouse (Fig. 3B). Endogenous *PpMYB8* expression was analyzed by RT–qPCR in samples from RNAi-*PpMYB8* plantlets to determine the extent of gene silencing compared with transgenic RNAi-EV control plants. As shown in Fig. 3C, significant differences in the relative abundance of the PpMYB8 transcripts were observed depending on the organ analyzed. *PpMYB8* down-regulation was stronger in primary needles than in secondary needles. Interestingly, in stem and roots, *PpMYB8* expression was decreased by ~80% and 60% compared with the reference level, respectively. The OE-*PpMYB8* lines were found to significantly overexpress *PpMYB8* in primary and secondary needles when compared with transgenic OE-EV controls [fold change (FC) from ~1.5 to 6; Fig. 3D]. However, much lower levels of overexpression were detected in the stem. No *PpMYB8* overexpression was observed in roots where paradoxically a certain degree of silencing was perceptible, particularly in the line 12PP22.

### Distinctive members of the *PpADT* gene family are down-regulated in RNAi-*PpMYB8* plants

**Fig. 4.**
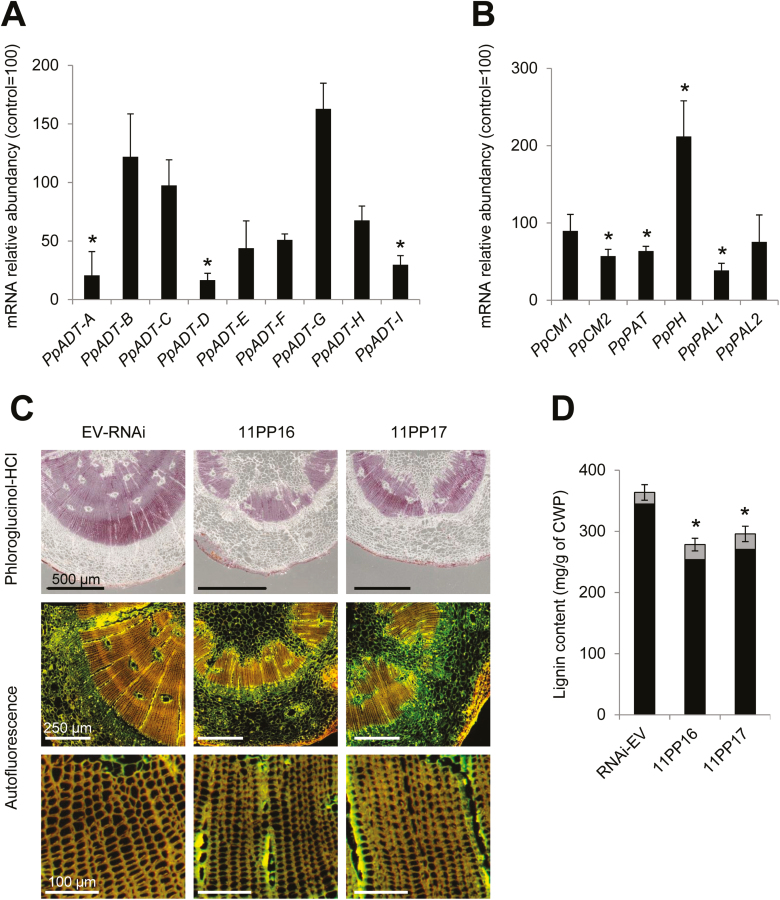
Effect of *PpMYB8* RNAi-mediated silencing in the regulation of Phe and phenylpropanoid biosynthesis and lignin deposition. (A) Average expression level of the nine ADT genes from *P. pinaster* in the RNAi*-PpMYB8* lines, expressed as a percentage relative to the RNAi-EV control. (B) Equivalent analysis to (A) for some key genes involved in Phe biosynthesis and the first steps of the biosynthesis of phenylpropanoids. (C) Vascular anatomy in cross-sections of stems from RNAi-*PpMYB8* transgenic plants (lines 11PP16 and 11PP17) and control plants (RNAi-EV). The first row shows phloroglucinol-HCl staining. The second and third rows show blue light autofluorescence. (D) Klason (black) and acid-soluble (gray) lignin determination in the stems from RNAi-EV control and RNAi-*PpMYB8* plants (11PP16, 11PP17). Gene abbreviations in (A) and (B) are as described in the legend of Fig. 2. Error bars represent the SD; asterisks indicate significant differences by *t*-test (α*=*0.01; *n*=3) between transgenic and control RNAi-EV lines.

To investigate to what extent *PpMYB8* regulates the expression of *ADT* genes during xylogenesis, the expression levels of the *ADT* genes were determined in the stems of the RNAi-*PpMYB8* lines (Fig. 4A). The silencing of *PpMYB8* resulted in a significant down-regulation of three *ADT* genes: *PpADT-A*, *PpADT-D*, and *PpADT-I.* Interestingly, *PpADT-A* and *PpADT-D* were among the most up-regulated *ADT* genes in CW (Fig. 2A), suggesting that PpMYB8 could directly, or indirectly, regulate the transcription of these particular *ADT* genes during xylogenesis. On the other hand, when the *ADT* expression profiles were also analyzed in the OE-*PpMYB8* plants, there were no significant differences between the transgenic plants and controls (Supplementary Fig. S1). The impact of *PpMYB8* silencing on the expression levels of other genes coding for enzymes directly involved in Phe biosynthesis was also determined. The results indicate a significant down-regulation of *PpPAT*, *PpCM2*, and *PpPAL1* in RNAi-*PpMYB8* plants, whereas the gene encoding phenylalanine hydroxylase (*PpPH)* was found to be overexpressed (Fig. 4B). The down-regulation of *PpPAT*, *PpCM2*, *PpPAL1*, and *ADT* genes is in good agreement with a potential role for PpMYB8 as a regulator of multiple genes involved in Phe biosynthesis and channeling of this amino acid into phenylpropanoids.

We hypothesized that down-regulation of genes related to the metabolism of Phe in the RNAi-*PpMYB8* transgenic lines could alter lignin content and/or structure, potentially affecting the formation of secondary cell walls, and therefore the formation of the tracheary elements of the xylem. Wiesner staining (phloroglucinol-HCl), which reveals the presence of lignin in a quantitative manner, and lignin autofluorescence after excitation under blue light showed that the RNAi-*PpMYB8* plants were strongly affected in their xylematic tissues (Fig. 4C). A severely thinned and irregular, discontinuous xylem cylinder was observed in the stems from these plants. In addition, individual tracheids in the stems of RNAi-*PpMYB8* plants were also observed to be narrower than in control plants (Fig. 4C, lower panels).

To further determine the consequences of *PpMYB8* down-regulation on lignin deposition, acid-soluble and insoluble (Klason) lignin contents were estimated in the crude cell wall residue from stems of RNAi-*PpMYB8* plantlets. The silenced lines exhibited an ~30% decrease in the total lignin content (acid-soluble+acid-insoluble lignin) when compared with the control plants (Fig. 4D), confirming previous anatomical observations (Fig. 4C). In addition, the ratio of the H- and G-derived monomers of lignin was evaluated by thioacidolysis, demonstrating a significant decrease in the H:G ratio in the RNAi-*PpMYB8* plants (Supplementary Table S1).

### The 5'-flanking regions of the *PpADT* genes contain putative R2R3-MYB-binding sites

Considering the current lack of a whole-genome assembly for *P. pinaster*, the reference genome of the closely related conifer *P. taeda* (V1.01 Genomic Scaffolds: https://pinerefseq.faculty.ucdavis.edu/; Wegrzym *et al*., 2014) was searched to identify the 5'-flanking regions of the *ADT* genes and used as a template to identify and assemble their *P. pinaster* relatives (SustainPineDB: http://www.scbi.uma.es/sustainpine/) (Supplementary Fig. S2). The length of the sequences assembled ranged from a minimum of 517 bp for *PpADT-B* to a maximum of 4804 bp for *PpADT-E*. *In silico* analysis of the *PpADT* 5'-flanking regions showed the presence of putative R2R3-MYB-binding sites in all of these sequences, with the single exception of *PpADT-B* and *PpADT-H.* Interestingly, this analysis also revealed the presence of several ACGT-based motifs in most of these 5'-flanking regions, which are the consensus binding sites for bZIP TFs (Deppmann *et al.*, 2006).

**Fig. 5.**
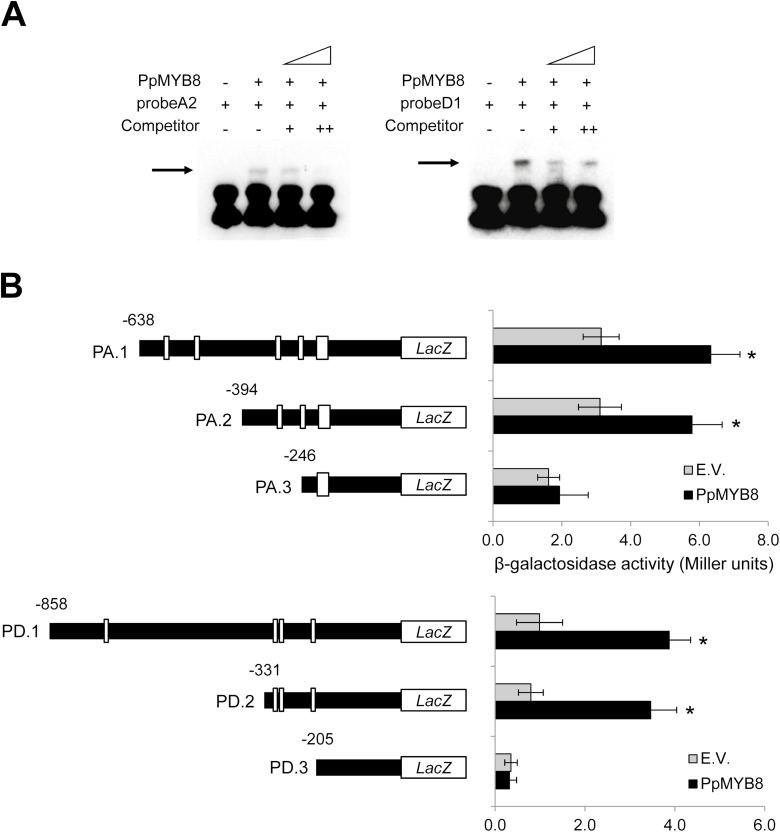
PpMYB8 interacts with putative regulatory regions of *PpADT-A* and *PpADT-D.* (A) EMSAs using recombinant PpMYB8 and the nucleotide probes A2 and D1 that contain potential AC-binding elements of the 5'-upstream regulatory region of *PpADT-A*, and *D*, respectively (see Supplementary Fig. S3). Band shifting after the formation of a probe–PpMYB8 complex is indicated with a black arrow. Lane 1, probe without PpMYB8; lane 2, probe and PpMYB8; lanes 3 and 4, probe, PpMYB8, and unlabeled probe as competitor. (B) β-Galactosidase reporter assay in yeast using subsequent deletions from regulatory regions of *PpADT-A* (PA.1, .2, and .3) and *PpADT-D* (PD.1, .2, and .3). White boxes indicate candidate AC-binding elements. Gray bars indicate β-galactosidase background activity (strains transformed with an empty construct, EV); black bars show β-galactosidase activity in strains transformed with the pDEST22-*PpMYB8* yeast expression construct. Error bars represent the SD; asterisks indicate significant differences by *t*-test (α*=*0.01; *n*=3).

A close-up analysis of these 5'-flanking sequences showed a particular enrichment of putative R2R3-MYB-binding sites in the proximal region of *PpADT-A* and *PpADT-D* (Fig. 5; Supplementary Fig. S2). The 5'-flanking region of *PpADT-A* (*P*_*ADT-A*_) analyzed contains 1907 bp upstream of the predicted translation start codon, presenting three AC elements in this region at positions 301 (AC-III element), 247 (AC-II element), and 209 (tandem repeated AC-II element) (see Gómez-Maldonado *et al.*, 2004 for a description of these AC elements). The respective 5'-flanking region of *PpADT-D* (*P*_*ADT-D*_) comprises 1226 bp from the predicted translation start codon, with three AC-II class elements in its proximal region at positions 298, 283, and 206.

### PpMYB8 is able to bind *PpADT-A* and *PpADT-D* regulatory regions

To investigate whether the putative *cis*-AC elements found in the 5'-upstream regions are important for PpMYB8 recognition, we used an EMSA to establish a physical link between PpMYB8 and synthetic DNA probes from *P*_*ADT-A*_ and *P*_*ADT-D*_. The following probes were designed as follows: for *P*_*ADT-A*_, probe A1 from position 225 to 329 (105 bp) and probe A2 from 281 to 370 (90 bp); for *P*_*ADT-D*_, probe D1 from 253 to 331 (66 bp) and probe D2 from 192 to 318 (127 bp) (Supplementary Fig. S3). Band shifting was observed for probe A2 and probe D1 in the presence of recombinant PpMYB8 protein (Fig. 5A). The band shift was reduced (probes A2 and D1) when unlabeled competitor DNA was added. In contrast, no shift was detected for probe A1 and probe D2 fragments. These results point to the existence of a physical interaction between PpMYB-8 and the putative AC-binding elements included in these probes.

To confirm an *in vivo* direct interaction between PpMYB8 and the putative regulatory regions *P*_*ADT-A*_ and *P*_*ADT-D*_, a β-galactosidase reporter assay was performed in the yeast *S. cerevisiae*. For *P*_*ADT-A*_ the following 5'-sequential deletions were assayed: 638 (PA.1), 394 (PA.2), and 246 (PA.3) (Fig. 5B). A significant increase in β-galactosidase activity in the presence of the prey construct was detected for the baits PA.1 (638) and PA.2 (394) compared with controls. In contrast, β-galactosidase activity was strongly reduced in yeast cells carrying the PA.3 (246) deletion. These results are consistent with previous EMSAs, indicating that AC elements located at 301 and 247 play a critical role in the transcriptional regulation of *PpADT-A* by PpMYB8. The following bait constructs were designed to analyze the regulatory properties of *P*_*ADT-D*_: PD.1 (858), PD.2 (331), and PD.3 (205). Similarly, as found for *P*_*ADT-A*_, the AC-II-binding elements located at positions 298, 283, and 206 of the 5'-flanking region of *P*_*ADT-D*_ were found to be critical for the recognition by PpMYB8, as demonstrated by the drastic reduction of β-galactosidase activity in PD.3 (Fig. 5B).

### Transcriptional reprogramming induced by silencing of *PpMYB8*

**Fig. 6.**
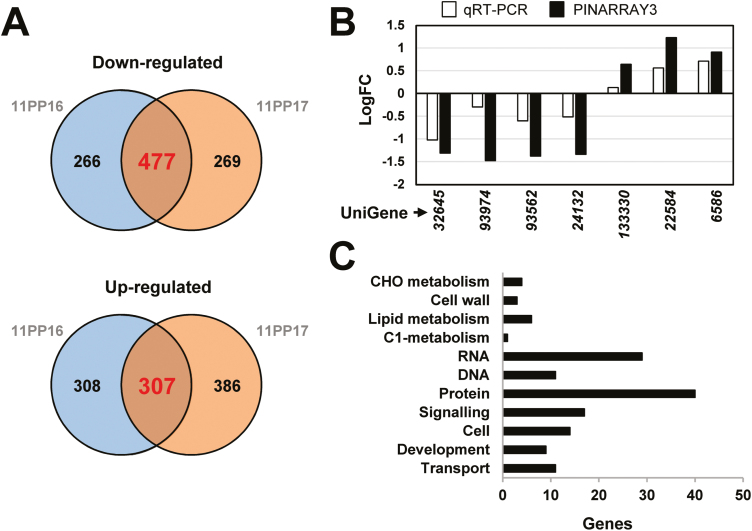
Transcriptome analysis of RNAi*-PpMYB8* plant stems. (A) Venn diagram showing both unique and overlapping (bold) expressed genes of significantly up-regulated and down-regulated genes between transgenic lines 11PP16/11PP17 and control plants (empty vector). (B) Validation of microarray results by RT–qPCR. Fold changes (logFC) of gene expression in control and RNAi-*PpMYB8* lines (mean of 11PP16 and 11PP17) analyzed using PINARRAY3 (black bars) and qPCR (white bars) are shown. (C) Enrichment analysis of functional categories (MapMan/Mercator web tool). The horizontal bars represent the number of genes included in each functional category.

To identify the transcriptional network under the control of PpMYB8 in more depth, we performed transcriptomic analysis of RNAi-*PpMYB8* plants. A 60-mer oligonucleotide microarray (PINARRAY3) developed in our laboratory and manufactured by Agilent (Santa Clara, CA, USA; Cañas *et al.*, 2015) was used. Twelve microarray fields were hybridized with four biological replicates corresponding to stems from RNAi-EV and four replicates of each of the RNAi-*PpMYB8* transgenic lines 11PP16 and 11PP17. Taken together, our analysis indicated 784 spots considered to represent DEGs (adjusted *P*-value <0.05; logFC>0.5). Among these genes, 307 were up-regulated in plants silenced for *PpMYB8* as compared with controls, whereas 477 were down-regulated (Fig. 6). Among the down-regulated genes, we identified a broad set of TFs including two MYBs, (*sp_v3.0_unigene19258* and *sp_v3.0_unigene3263*), a bZIP (*sp_v3.0_unigene8197*), a RAV/NGATHA (*sp_v3.0_unigene34155*), a MADS box (*sp_v3.0_unigene32645*), a C3H zinc finger (*sp_v3.0_unigene17894*), a GARP (*sp_v3.0_unigene19789*), and a bHLH (*sp_v3.0_unigene29985*). Consistent with the strong effect on xylem tissue in plants silenced for *PpMYB8*, we detected down-regulation of genes directly involved in cell wall modification such as xyloglucan endotransglucosylase/hydrolase (*sp_v3.0_unigene35083*), a wall polysaccharide-specific *O*-acetyltransferase (*TBL*-*TRICHOME BIREFRINGENCE-LIKE*, *sp_v3.0_unigene7027*), a pectinesterase (*sp_v3.0_unigene9201*), an oxidoreductase (*sp_v3.0_unigene2867*), a chitinase (*sp_v3.0_unigene29478*), and up to three *S*-adenosyl-l-methionine-dependent methyltransferases putatively involved in cell wall biogenesis (*sp_v3.0_unigene1437*, *sp_v3.0_unigene96035*, and *sp_v3.0_unigene93562*). Also consistent with the observed phenotype, multiple genes with a role in plant development were down-regulated, including *NO VEIN* (*sp_v3.0_unigene63788*), the actin cytoskeleton organizer *PIROGI* (*sp_v3.0_unigene13374*), and the mRNA decapping DCP2 (*sp_v3.0_unigene183375*) whose silencing in Arabidopsis results in disorganized veins. Additionally, an important group of genes involved in cell organization and division were also down-regulated including *PROHIBITIN 3* (*sp_v3.0_unigene24132*), *CRUMPLED LEAF* (*sp_v3.0_unigene1324*), and multiple cytoskeleton-related genes (*sp_v3.0_unigene5018*, *sp_v3.0_unigene93974*, *sp_v3.0_unigene13374*, *sp_v3.0_unigene2327*, and *sp_v3.0_unigene6084*) (Supplementary Table S4).

Among the up-regulated genes, we detected several genes related to protein post-translational modification, protein degradation, development, and cell organization (Supplementary Table S4). Additionally, the set of up-regulated genes included further TFs putatively involved in development and differentiation processes such as an MYB (*sp_v3.0_unigene133569*) orthologous to the Arabidopsis *AtMYB35*, *DEFECTIVE IN MERISTEM DEVELOPMENT AND FUNCTION 1*, a *TCP* putatively orthologous to the Arabidopsis *PLASTID TRANSCRIPTION FACTOR 1* (*PTF1*) involved in chloroplast regulation of leaf differentiation (*sp_v3.0_unigene6586*), and a *HIGH MOBILITY GROUP* (*HMG*) box protein (*sp_v3.0_unigene24902*). Interestingly, we also detected up-regulation of tryptophan synthase (*sp_v3.0_unigene133330*) and enoyl-CoA hydratase/isomerase D (*sp_v3.0_unigene22584*), key enzymes involved, respectively, in the biosynthesis of Trp and vitamin K1, through two alternative pathways that, as with the Phe/Tyr pathway, start from chorismate. Also related to amino acids, two additional up-regulated genes were detected, an ACT domain repeat protein (*sp_v3.0_unigene8593*), involved in feedback regulation of amino acid metabolism, and ornithine delta-aminotransferase (*sp_v3.0_unigene5775*), involved in arginine catabolism. The microarray results were validated by RT–qPCR analysis of a set of DEGs (Fig. 6B). A functional enrichment analysis, using the Mapman categories through the Mercator web tool, was performed, revealing a set of categories with significant differences between RNAi-*PpMYB8* plants and controls, including, among others, Cell wall, Development, Signaling, or Cell organization (Fig. 6C). We also analyzed the transcriptome of stems from OE-*PpMYB8* plants to characterize the putative impact of *PpMYB8* overexpression. In contrast to what was observed in RNAi-*PpMYB8* plants, little impact was observed as a consequence of *PpMYB8* overexpression (Supplementary Table S5).

### PpHY5 interacts with the regulatory regions of *PpADT-A* and *PpADT-D*

**Fig. 7.**
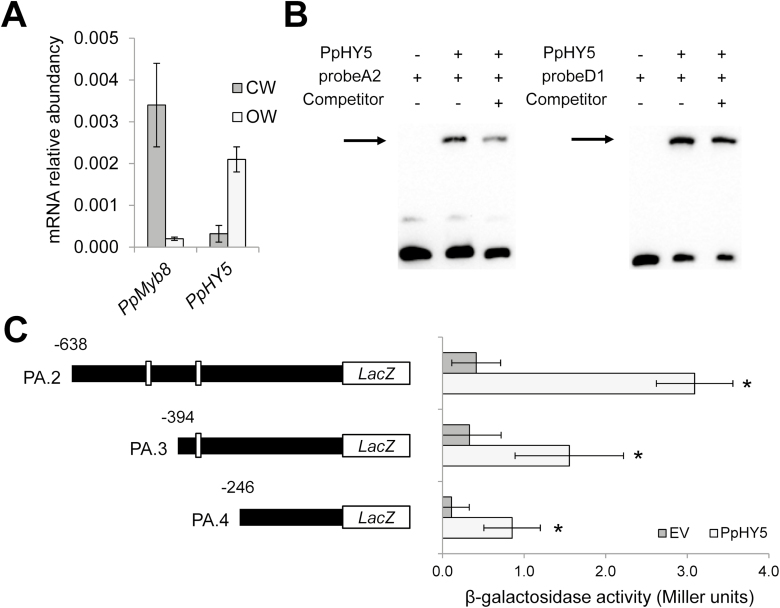
PpHY5 is able to bind to the *PpADT-A* and *PpADT-D* putative regulatory regions. (A) Expression pattern of PpMYB8 versus PpHY5 in CW and OW. (B) EMSAs using recombinant purified PpHY5 protein and nucleotide probes probeA2 and probeD1 (the same as present in Fig. 5A). (C) β-Galactosidase reporter assay in yeast using subsequent deletions of the putative promoter of *PpADT-A* (PA.1, .2, and .3). White boxes indicate the location of ACGT elements within the 5'-upstream sequence. Background β-galactosidase activity is represented in dark gray. Bars in pale gray represent β-galactosidase activity in yeast strains transformed with the pDEST22-*PpHY5* yeast expression construct. Error bars represent the SD; asterisks indicate significant differences by *t*-test (α*=*0.01; *n*=3).

Microarray analysis of *PpMYB8-*silenced plants showed that in the 11PP16 line, *PpHY5* (*sp_v3.0_unigene6404*), a pine ortholog of Arabidopsis *HY5* (*ELONGATED HYPOCOTYL 5*), was significantly up-regulated (adjusted *P*-value=0.04) while, in the 11PP17 line, the adjusted *P*-value was 0.09. HY5 is a central TF that promotes, among other processes, the accumulation of Phe-derived compounds and the repression of critical genes for cell wall biogenesis. Moreover, there is evidence demonstrating the coordination of HY5 and MYB TFs in different processes related to the synthesis of Phe-derived compounds (Shin *et al.*, 2013; Nguyen *et al.*, 2015; Czemmel *et al.*, 2017); on the other hand, our previous observation revealed the existence of multiple potential binding sites for HY5 (ACGT elements) in the 5'-flanking regions of most PpADTs (Supplementary Fig. S3). Thus, we decided to investigate whether this gene might be associated with lignin accumulation in pine by analyzing its expression in CW versus OW. In this regard, RT–qPCR analysis demonstrated that, unlike what was observed for *PpMYB8*, the expression of *PpHY5* in CW is much lower than it is in OW (Fig. 7A).

The upstream regions of *PpADT-A* and *PpADT-D* are particularly enriched in ACGT elements, so we performed EMSAs using recombinant PpHY5 protein to test a putative interaction. Our results revealed that recombinant PpHY5 can bind to *PpADT-A* and *PpADT-D* gene promoters in a manner similar to PpMYB8 (Fig. 7B). In addition, a β-galactosidase assay in yeast confirmed that ACGT boxes located in *ADT-A* at positions 347 and 464 contribute to PpHY5 recognition in an accumulative manner (Fig. 7C).

## Discussion

The biosynthesis of lignin in trees requires a massive supply of its essential precursor Phe. Therefore, the biosynthesis of this amino acid requires fine regulation presumably at the transcriptional and post-transcriptional levels. In the present work, we have investigated the transcriptional regulation of ADT enzymes related to lignification in *P. pinaster*. The use of a conifer tree to perform these studies is particularly relevant, due to the quantitative importance of lignin biosynthesis in these plants. The role of ADTs could be especially relevant, as these enzymes have been proposed to be a rate-limiting step of Phe biosynthesis (Maeda and Dudareva, 2012). Moreover, the existence of a single *PAT* gene in most plants and multiple *ADT* isogenes strongly suggests that different ADT isoforms could be involved in the biosynthesis of Phe for different and specific metabolic fates. Consistently, Corea *et al.* (2012) used different combinations of *ADT* knockouts in *Arabidopsis thaliana* and found that the six Arabidopsis ADT isoforms differ profoundly in their contribution to lignin accumulation, with AtADT5 being the most important contributor.

The comparative study of CW and OW in conifers provides an excellent model for dissecting the regulatory cues that coordinate metabolic pathways providing the substrates for lignification and xylogenesis. CW is characterized by the deposition of a thicker secondary cell wall, with more lignin content, which is structurally enriched in *p*-hydroxyphenylpropane units (H-lignin) and reduced levels of cellulose (Groover, 2016). Previous studies have also shown that the development of CW is correlated with the up-regulation of genes coding for enzymes involved in the construction of the secondary cell wall (Villalobos *et al.*, 2012). To identify *PpADT* genes putatively involved in the biosynthesis of Phe for the later biosynthesis of lignin, we compared the expression of these genes in CW and OW. Our results showed an increased expression in CW of six out of the nine *ADT* genes in maritime pine (Fig. 2A). However, *PpADT-A* and, to a lesser extent, *PpADT-G* and *PpADT-D*, were the most highly expressed genes. At the same time, a set of genes directly linked to Phe metabolism, those encoding the chorismate mutase isoforms 1 and 2 (*PpCM1* and *PpCM2*), prephenate aminotransferase (*PpPAT*), phenylalanine hydroxylase (*PpPH*), and phenylalanine ammonia lyase (*PpPAL1*), were up-regulated in CW compared with OW (Fig. 2B).

Previous studies have supported a role for MYB8 as a key transcriptional regulator of phenylpropanoid metabolism and secondary cell wall biogenesis in conifers (Bomal *et al.*, 2008; Craven-Bartle *et al.*, 2013). Accordingly, the heterologous overexpression of *PtMYB8* from *P. taeda* in young plantlets of *P. glauca* resulted in the up-regulation of a set of genes involved in the biosynthesis of monolignols and the parallel down-regulation of other genes putatively involved in the same pathway (Bomal *et al.*, 2008). These authors further reported the reduced root growth phenotype of *in vitro* transgenic plantlets that did not survive to subsequent transfer to soil. However, in the present work, it has been found that the homologous overexpression of *PpMYB8* in maritime pine did not affect the phenotype of the 16-month-old transgenic plants, and it consistently exhibited only small changes in the transcriptome (Fig. 3B; Supplementary Table S5). These results indicate that an increase in the endogenous levels of this TF does not have a relevant effect on the regulation of its target genes, including *ADT* genes. In addition, we gained little evidence in maritime pine for adverse effects of *MYB8* overexpression on somatic embryo production and conversion into rooted somatic seedlings or growth of trangenic plants after 12 and 24 months (Fig. 3B). In contrast, *P. pinaster* plantlets silenced for *PpMYB8* exhibited concomitant down-regulation of three ADTs: PpADT-A, PpADT-D, and PpADT-I (Fig. 4A). Interestingly, these isoforms were transcriptionally activated in CW compared with OW, an effect that was particularly strong for *PpADT-A* and PpADT-D (Fig. 2A). Additional genes linked to Phe metabolism were also down-regulated, with the exception of *PpPH*, whose expression was enhanced in the silenced plants (Fig. 4B). This finding suggests that Phe hydroxylation is not associated with the metabolic activity of the ADT-A and ADT-D isoforms.

The RNAi-*PpMYB8* lines exhibited a significant decrease in growth, reduced levels of lignin, and an altered H-:G-lignin ratio (Fig. 3A; Supplementary Table S1). Consistently, the silencing of *PpMYB8* was observed in different organs, with particularly reduced levels of transcripts in the stems of the transgenic lines (Fig. 3C). Microscopic anatomy of the stems and histochemical detection of lignin support the hypothesis that *PpMYB8* down-regulation hampers the proper development of the xylem, significantly reducing lignin deposition and presumably affecting plant growth (Fig. 4C, D). It is still unclear whether a fine-tuning in *PpMYB8* down-regulation could result in a reduction in lignin content without severely affecting the overall plant growth rate.

The marked developmental alterations observed in RNAi-*PpMYB8* plants strongly point to a significant underlying modification of the plant transcriptome. In this regard, we investigated whether the observed down-regulation of certain *PpADT* genes in these plants occurs through the direct activity of PpMYB8 on the corresponding promoters, or whether the reduction observed in their expression was a consequence of a pleiotropic effect. In this regard, *in silico* analysis of the 5'-upstream regions of the pine *ADT* genes revealed the occurrence of putative R2R3-MYB-binding sites in all of them, with the exception of those corresponding to *PpADT-B* and *PpADT-H* (Supplementary Fig. S2). This result is consistent with the broadly characterized regulation of phenylpropanoid biosynthesis in plants through MYB TFs (Zhong and Ye, 2007; Nakano *et al.*, 2015; Ye and Zhong, 2015; Lamara *et al.*, 2016). The particular enrichment of R2R3-MYB-binding sites in the 5'-flanking regions of *PpADT-A* and *PpADT-D* mimics the previously described promoter architecture and PpMYB8 regulation of *PpPAT* and *PpPAL1* genes coding, respectively, for enzymes catalyzing the preceding and subsequent reactions of ADT (Craven-Bartle *et al.*, 2013). These observations strongly suggest that these ADT isoforms are involved in the lignin-associated Phe biosynthetic pathway in maritime pine. To determine whether the putative regulatory regions of PpADT-A and PpADT-D physically interact with PpMYB8, a complementary strategy using EMSAs and gene reporter assays in yeast was carried out. Both approximations have provided concurrent evidence about the direct regulation of *PpADT-A* and *PpADT-D* expression by PpMYB8 through the recognition of a region enriched in AC elements (Fig. 5A, B).

As we have shown, silencing of *PpMYB8* resulted in a large alteration of plant growth and development. These findings suggest that the *RNAi* silencing of *PpMYB8* could be triggering a complex combination of direct and indirect alterations of the stem transcriptome. To further identify other genes putatively co-regulated with *PpADT-A* and *PpADT-D* and also with *PpPAT* and *PpCM2*, in the lignin-associated Phe biosynthesis pathway, the transcriptomes of the RNAi-*PpMYB8* plantlets were analyzed. Consistent with our hypothesis, we identified a large number (784) of DEGs compared with the controls that were shared by both the 11PP16 and 11PP17 RNAi-*PpMYB8* lines, of which 307 genes were up-regulated and 477 were down-regulated (Fig. 6). Markedly, we detected that the silencing of *PpMYB8* results in the parallel down-regulation and up-regulation of a complex network of TFs belonging to different families: R2R3-MYBs, bZIP, RAV/NGATHA, MADS box, C3H zinc finger, GARP, HLH TCP, or HMG-Box, suggesting that a significant proportion of the DEGs are regulated in this process through TFs other than PpMYB8. Interestingly, the down-regulated GARP TF belongs to a family of TFs related to the MYB superfamily that have been suggested to be central coordinators of plant nutrition and development (Safi *et al.*, 2017), so its putative involvement in the channeling of nitrogen resources towards the biosynthesis of Phe and later synthesis of lignin is of particular interest.

Interestingly, among the down-regulated DEGs, we have detected several genes coding for enzymatic activities directly related to cell wall modification including xyloglucan endotransglucosylase/hydrolase, pectinesterase, chitinase, wall polysaccharide *O*-acetyltransferase, and oxidoreductases, and up to three *S*-adenosyl-l-methionine-dependent methyltransferases putatively involved in monolignol biosynthesis. Down-regulation of these activities could be associated with an altered biogenesis of the secondary cell wall in stem cells that, in turn, results in disorganization of the xylematic tissues (Fig. 4C). Consistently, we also found down-regulation of multiple genes directly associated with the architecture of the cytoskeleton, which could be directly related to the same process of xylem disorganization. In this context, it should be mentioned that there was down-regulation of a gene orthologous to the Arabidopsis *NO VEIN*, encoding a plant-specific nuclear factor required for leaf vascular development (Tsugeki *et al.*, 2009), and we hypothesize that its deregulation may be directly involved in the altered phenotype of vascular development in pine stems.

Among the up-regulated genes, we have detected two genes that encode key enzymes participating in the biosynthetic pathways of Trp and vitamin K1: tryptophan synthase and enoyl-CoA hydratase/isomerase D. Both pathways use chorismate as a precursor and therefore compete with the Phe/Tyr pathway for its use. We have shown that the silencing of PpMYB8 involves down-regulation of important genes involved in the Phe pathway so presumably, as a result, the availability of chorismate increases and could be channeled towards the synthesis of Trp and vitamin K1. Additionally, Trp is a precursor for the biosynthesis of auxin, a hormone related to cell elongation, and in this regard we have identified up to seven auxin-related genes putatively involved in functions such as hormone transport, vascular pattern, gravitropism, and dormancy (Supplementary Table S4). These results suggest that a set of auxin-related genes are transcriptionally activated in response to molecular cues associated with cell elongation defects triggered by the silencing of *PpMYB8*.

The observed up-regulation of an ACT domain repeat (ACR) protein is particularly interesting since these proteins are involved in feedback regulation of amino acid metabolism and function in modulating the activity of amino acid biosynthetic enzymes (Singh *et al.*, 2018). For example, in Arabidopsis, *AtACR11* is coordinately expressed with the gene encoding chloroplastic glutamine synthetase (GS2), and the AtACR11 protein activates GS2 activity (Singh *et al.*, 2018). In this regard, *sp_v3.0_unigene8593* encodes the ortholog of Arabidopsis *AtACR4*, a gene strongly co-expressed with that coding for tryptophan aminotransferase (http://atted.jp/), which is essential for auxin biosynthesis, and thus this supports the possible involvement of auxin in the RNAi-*PpMYB8*-mediated phenotype.

As previously mentioned, the biosynthesis of Phe in plants is associated with multiple processes of enormous physiological importance that therefore requires fine and complex regulation at different transcriptional, post-translational, and metabolic levels. Thus, we have demonstrated here that during xylogenesis, PpMYB8 plays a fundamental role in the transcriptional regulation of several important genes involved in this process. However, the participation of these enzymes in the synthesis of Phe associated with other metabolic processes suggests that the corresponding genes may have alternative mechanisms of transcriptional regulation through the action of other TFs or combinations of TFs. In this work, we provide experimental data suggesting that the expression levels of *PpADT-A* and *PpADT-D* are also regulated by PpHY5. We have shown that this TF is able to bind the regulatory regions of *PpADT-A* and *PpADT-D*, and also that the expression profile of this gene is opposite to that of PpMYB8 in CW, OW, and in RNAi-*PpMYB8* plants, suggesting an antagonistic role. Functional studies in transgenic plants are required to address whether PpHY5 could be repressing the expression of *PpADT-A* and *PpADT-D* in competition with activator TFs such as PpMYB8.

In summary, our results support the hypothesis that distinctive members of the *ADT* gene family are transcriptionally regulated by PpMYB8 for the provision of precursors for the synthesis of lignin that is necessary for the proper development of xylematic tissues in maritime pine as previously proposed (Craven-Bartle *et al.*, 2013). Recently, we have shown that PpNAC1 is a central regulator governing secondary cell wall formation, and thus Phe biosynthesis and utilization, in *P. pinaster* (Pascual *et al.*, 2018). In that work, we also demonstrated that PpNAC1 partially mediates its activity through direct activation of PpMYB8. Future research will allow for deepening our knowledge of the complementary or alternative regulatory mechanisms that govern Phe biosynthesis dependently and independently of PpMYB8 and PpNAC1 in this conifer species.

## Supplementary data

Supplementary data are available at *JXB* online.

Table S1. Estimated H- and G-lignin content in RNAi-*PpMYB8* silenced lines compared with the control (RNAi-EV).

Table S2. Primer sequences used for cloning.

Table S3. Primer pairs used for RT-qPCR.

Table S4. Transcriptome analysis of transgenic plants silenced for *PpMYB8* (RNAi- *PpMYB8*).

Table S5. Transcriptome analysis of transgenic plants overexpressing *PpMYB8* (OE-*PpMYB8*).

Fig. S1. Average expression level of the nine ADT genes from *P. pinaster* in the OE*-PpMYB8* lines, expressed as a percentage of controls

Fig. S2. *In silico* analysis of candidate AC- and bZIP-binding elements within the 5'-flanking region of the ADT genes in *Pinus pinaster*.

Fig. S3. Probes designed for the EMSA in the *P*_*ADT-A*_ and *P*_*ADT-D*_ sequences.

Protocols S1.

eraa099_suppl_Supplementary_table_S1-S3_figure_S1-S3

eraa099_suppl_Supplementary_data_1

eraa099_suppl_Supplementary_data_2

eraa099_suppl_Supplementary_protocols
