# Supplementary material for: Transcriptional analysis of arogenate dehydratase genes identifies a link between phenylalanine biosynthesis and lignin biosynthesis
Source: J Exp Bot. 2020 Feb 24;71(10):3080–93. doi: 10.1093/jxb/eraa099 (PMC7260716; doi:10.1093/jxb/eraa099)
Supplement: eraa099_suppl_Supplementary_table_S1-S3_figure_S1-S3 [file eraa099_suppl_supplementary_table_s1-s3_figure_s1-s3.pdf]

Table S1. Estimated H- and G-lignin content in RNAi-*PpMYB8* silenced lines compared to control (RNAi-EV). Asterisk indicates significant differences between RNAi-PpMYB8 plants and controls (*P* = 0.032, *n* = 3).

sample	%H-monomers	%G-monomers
RNAi-*PpMyb8*	1.5 ± 0.3%*	98.5 ± 0.3% *
RNAi-EV	2.1 ± 0.2%	97.9 ± 0.2 %

Table S2. Primer sequences used for cloning.

Primer name	Primer sequence (5'→3')
p1830.5Fwd	ATGATCCAAGAAATGCAAGTCACTC
p1830.7Rvs	TCTACAGATATCGATGAGTCTCG
p1830.4Fwd	TTAGGGTATTTTGACTCCTGTC
p1830.6Rvs	TTTTCTGGAATTTGAGGTTTAC
p3030.2Fwd	GTACAACACTTGATTAAGGTTGCG
p3030.2Rvs	CTTGGAATCGTCGCTGTTGCTTC
p61682.2Fwd	CATAGAACTTCACATCGCTTCC
p61682.2Rvs	AAAGGCACATCTCAGAGCC
PA1 AttB4	ATAGAAAAGTTGGATTCCACTTAGGACTCCACCCC
PA2 AttB4	ATAGAAAAGTTGGAGCCACAGACGATGATGACCCA
PA3 AttB4	ATAGAAAAGTTGGAACCCTAGATCAGCATGAGCTA
pA AttB1R	TTGTACAAACTTGCTTTTCTGGAATTTGAGGTTTAC
pD1 AttB4	ATAGAAAAGTTGGAGTACAACACTTGTTAAGGT
pD2 AttB4	ATAGAAAAGTTGGACCAGGGTGGGAAAGCTGGTG
pD3 AttB4	ATAGAAAAGTTGGAATGGGATGGAGCAGAATATATAC
pD AttB1R	TTATACAAACTTGAATTGGAATCGTCGCTGTTGC
PI1 AttB4	ATAGAAAAGTTGGACTTCCATAAATATTGAAACAC
PI2 AttB4	ATAGAAAAGTTGGAGATTGGCAAGATGGATAAAG
PI3 AttB4	ATAGAAAAGTTGGACAAAACTAGATTGAGAAAGG
PI AttB1R	TTGTACAAACTTGCAGCCTTGTAGATAATTAGTG
AttB4	GGGGACAACTTTGTATAGAAAAGTTG
AttB1R	GGGGACTGCTTTTTTGTACAAACTTG
MYB8 AttB1	AAAAAGCAGGCTTAATGGGGCGCCACTCGTG
MYB8 AttB2	AGAAAGCTGGGTTCTAAATTTGGTCCAGAAC
AttB1	GGGGACAAGTTTGTACAAAAAAGCAGGCT
AttB2	GGGGACCACTTTGTACAAGAAAGCTGGGT

Table S3. Primers pairs used for RT-qPCR.

Gene name	Gene description	Forward / Reverse primers sequences (5'→3')
PpADT-A	Arogenate dehydratase-A	GACTATACTTCATGTACCAAGC / CCACGATCTTCAATATCTCTGG
PpADT-B	Arogenate dehydratase-B	AGTGACATGGGGAAGTCACC / CGATACACATATTTCCTCTCTAC
PpADT-C	Arogenate dehydratase-C	ATGAAGGCGTGATTTGAAGC / ACTCTGCCACAGAGGGACAC
PpADT-D	Arogenate dehydratase-D	CGGTGAAAATAAGAGACTGGG / TGTATATGACCAGTAGCTGCTC
PpADT-E	Arogenate dehydratase-E	CAAATCCGTGTTCTCTGCTGG / TGAGGATCCTGAAGTTCCACG
PpADT-F	Arogenate dehydratase-F	CTCCGTTCTTGTGACAGCTCG / TTTGATCCTCAAATCCCTGTAC
PpADT-G	Arogenate dehydratase-G	GTTCATGTAACTGAGAACTGGG / AACTTAGTGTCACACCCTCTC
PpADT-H	Arogenate dehydratase-H	CAGTTGTATCAGAGGTATCGG / TGGCGTTGGCTAGATCCTTG
PpADT-I	Arogenate dehydratase-I	ATGTGTACGCATGTCTGTATAG / CCATGAAGATCCTCGCGATTG
PpCM1	Chorismate mutase-1	GAACTTCCATGGATATACCTGC / AGCAATTCAATTTGCTGACTGC
PpCM2	Chorismate mutase-2	GCCGTTAAATTGTTGTGTCTACC / TGATTTTCCCGAACCAGAAG
PpPAT	Prephenate/arogenate aminotransferase	GCAGTCATCATTGCCGAAGGC / AGATTGCAAGCCATGGAGGG
PpPH	Phenylalanine hydroxylase	AGCTATTGGAATCACCTCACC / GGCAATATCCCTCATCTTGTC
PpPAL1	Phenylalanine ammonia lyase-1	GATTTGCATCCCACTGGATT / AAGCAGTACACGATCACCCA
PpPAL2	Phenylalanine ammonia lyase-1	GCAGCACGTTTTGCATAGAA / TAATATTTCCCAACACCGGC
PpMYB8	MYB8	CCACACGCAAGAGGAGACACTG / GGCGGAATTTAGGGCAGCGTGG
PpEF1α	Elongation factor 1α	TGCTGTTGGAGTCATCAAGG / CTCGTGCATCAGAATCAGACA
PpActin2	Actin	ATCTCTCAGCACATTCCAACAG / TATTGCCACCATCATCTCAA
sp_v3.0_unigene32645		TGGCTTCTCTTCCAATGCTC / ACCTGCTCAACCACTTCCAA
sp_v3.0_unigene93974		TGTAGGCATAAGAACTCCATCAA / CTGTGACAACTTCTCCGTGC
sp_v3.0_unigene93562		AAAACAGGGGCCTTACGAAG / ATGGTATATTGCAGGCAGGC
sp_v3.0_unigene24132		TGTGGTCTACTTGCCCTCTG / CGTGACTTGGGCTCATCATC
sp_v3.0_unigene133330		TGGCCAAAATCCCAAGTTTTCT / ACAGTCATATGTTCTTCCCACTT
sp_v3.0_unigene22584		TGGTTCAGAGGAGGCAAAAGA / AATAGGGCTGTGCTCCAACT
sp_v3.0_unigene6586		CAGAATCGCCCAGGGTATGA / ATGCGCGTAGAAAGAACACC

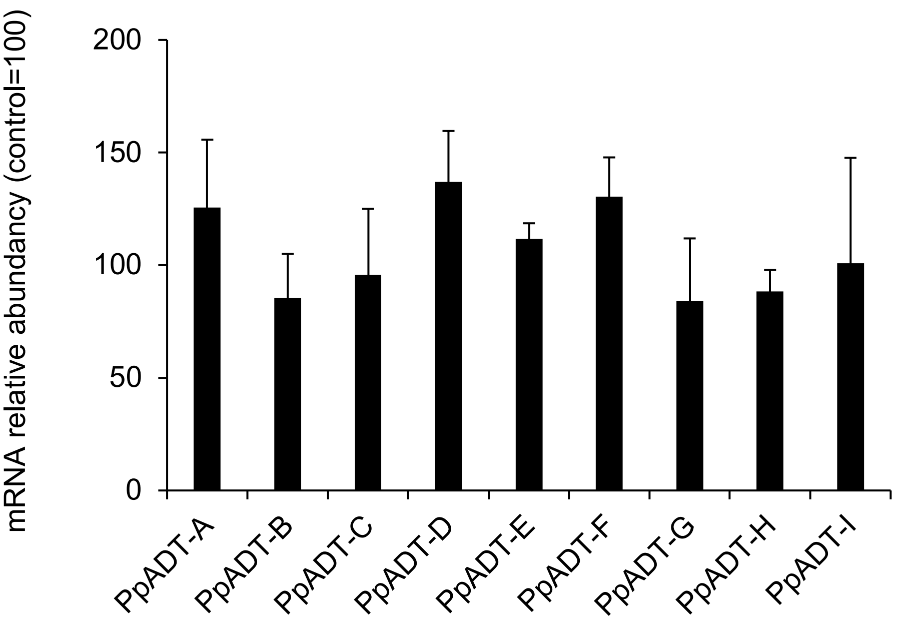

Figure S1. Average expression level of the nine *ADT* genes from *P. pinaster* in the OE-*PpMYB8* lines, expressed as percentage to controls.

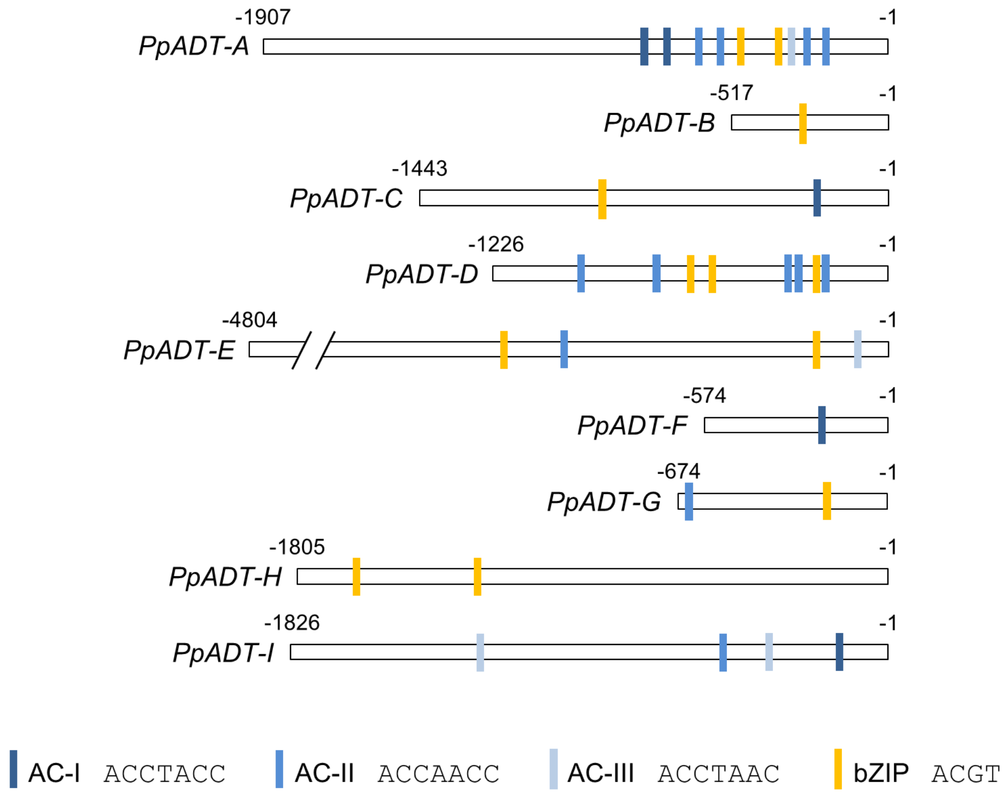

Figure S2. *In silico* analysis of candidate AC- and bZIP-binding elements within the 5'-flanking region of the *ADT* genes in *Pinus pinaster*. Genomic DNA sequences upstream of the initiation codon were manually assembled from the information available at *SustainPineDB*, using their *P. taeda* homologues as a reference. Different colours, as indicated in the key, were used to mark the three different AC-biding elements previously described as putative *PpMYB8* binding sites, and the putative ACGT bZIP binding sites.

PpADT-A proximal 5'-flanking region (*P*$P_{ADT\text{-}A}$)

TAGGAGACGACCCCACCTTTCAAGAAAAGGCTCTCATTAGAACACACTCCAACCAATCCCGGGC

CACCCCGGGGAAACCGGGTCTACGTAGACCGGTTCCCCGCCCCCTGAAAAGGCCAAAGTGCAAG

CATCAAAGACAAATGAGGTTCCACTTTTTGGCCACAGACGATGATGACCCAAGAACAACAGACT

probeA2

GGTAAGGCACACGTGCTTCCACGAGATCCACTAACTTGCGCGGGTGTTTACACACCTAACCACT

CCGGAGCTTCCATGCTTCATCCACGAGTCAGAGGGGCCTTTCCTCCAACCACCCTAGATCAGCA

probeA1

TGAGCTATTTTATCTACTACCCCTCCACCAACCCACCACCACCACCAAACAATCTTAACAGAAC

TCTCTATTAGTCTAAGCACGCCTACAAATGTTTTGCAGGCGAACCCAACTGGTTCGGACGAACG

AGTGTGTGATGTGCCCAAACACATACTCGCGTCCCAGATCTGAACACTCTGAGCCCTCGGCCCA

AATTTTCAGGGGGCTAAAGTAAACCTCAAATTCCAGAAAA**ATG**

PpADT-D proximal 5'-flanking region ($P_{ADT\text{-}D}$)

CTTTCTCATTCTGGTTTGGTAAGCACTTAACAAAAATTTCCAGATTGGACAGATAATTTTTGAG

TCCAACTAGGTTGTTACATTTGGCAGAGTAGATGGCAGGGTTGTGTAGAATGGTAAGTAAGGTG

GGGGAGGACAAAACTCAGTTTACTTGGTTTTCAATCCTGATGGTGCGCAGTCTCATTCCAAGTC

probeD1

TTAGATGCGTGGGGGCCAGGGTGGGAAAGCTGGTGAGAAGGGCCAAACCAAAAAAAAACCAAAC

GAAATCACAGCGTACGATCTAACTAACCGTGTTGAATAGTCCCGTGACGTAGCCGCCGTAATGT

probeD2

GCAACAACCCACCATGGGATGGAGCAGAATATATACAAAACAAGCAAAGGGTGGCCTCGCCTGC

TTCCTCTTTATTGTCATTGCCTTGCCAGCTTTGGAAAATCTTTAGCTTTGGAAAAATCTTTAGC

TCTGAGTGAAGAAAAGAGGACTGAACAGAAAGAGATTTGCAGCTGCAAATTTCAGGAGCAGTTA

TTTGAAGCAACAGCGACGATTCCAAG**ATG**

Figure S3. Probes designed for the EMSA in the $P_{ADT\text{-}A}$ and $P_{ADT\text{-}D}$ sequences. ATG codon is marked at the 3'-end. Putative AC-binding elements are marked in cyan; putative bZIP ACGT boxes are marked in yellow.
