# Supplementary material for: Transcriptional analysis of arogenate dehydratase genes identifies a link between phenylalanine biosynthesis and lignin biosynthesis
Source: J Exp Bot. 2020 Feb 24;71(10):3080–93. doi: 10.1093/jxb/eraa099 (PMC7260716; doi:10.1093/jxb/eraa099)
Supplement: eraa099_suppl_Supplementary_protocols [file eraa099_suppl_supplementary_protocols.pdf]

Supplementary protocols

Generation of overexpression and silenced transgenic lines of maritime pine for PpMYB8

A cryopreserved embryogenic line (PN519; Breton *et al.,* 2006; Trontin *et al.,* 2016) was used for genetic transformation and transgenic plant production. All transgenic lines and controls obtained or handled during this work were therefore derived from the same PN519 genetic background to facilitate data comparison. PN519 embryonal-suspensor masses (ESM) were reactivated from the cryopreserved stock following the method described by Harvengt (2005) but using a modified Litvay (Litvay *et al.,* 1985) basal formulation (mLV) as reported in Morel *et al.* (2014). Subsequently to thawing and prior to genetic transformation assays, ESM were subcultured for 6 weeks on fresh mLV multiplication medium (Morel *et al.,* 2014) using the plating method (Trontin et al. 2016) in order to stimulate a state of active ESM proliferation.

For *PpMYB8* over-expression, cDNA (accession FN868598) was integrated into the constitutive over-expression cassette (maize ubiquitine promoter; CAMV35S terminator) of vector pMBb7Fm21GW-UBIL (Ghent University, Belgium, Karimi *et al.,* 2002) to yield the OE-*PpMYB8* binary vector construct. For *PpMYB8* RNAi mediated silencing, a 243-bp-length fragment located at the 3'-end of a *Pinus taeda MYB8* EST (accession DQ399057, clone obtained from C. Plomion's lab, INRA, France) was amplified by PCR (forward: GCCTCCGTTTTCAAGCCACACGCA;reverse:GCAGCAATGCGGTGGAAGTCGGGA) and transferred into the Gateway® destination binary vector pB7GWIWG2(II) (NOS promoter and terminator; Karimi et al., 2002). In the resulting ihpRNA vector (named RNAi-PpMYB8), the 243 bp *MYB8* fragment was integrated as inverted repeats in the RNAi constitutive expression cassette. Both binary vectors, pMBb7Fm21GW-UBIL and pB7GWIWG2(II), harbored a *bar* gene cassette that allows effective selection of transgenic events with phosphinothricin in maritime pine (Trontin *et al.,* 2007; 2013).

The OE-*PpMYB8* and RNAi-*PpMYB8* binary constructs were transferred into the C58pMP90 strain of *A. tumefaciens* (Koncz and Schell, 1986) following standard protocols. *Agrobacterium*-mediated genetic transformation of the PN519 embryogenic line was performed using DCR-based media (Gupta and Durzan, 1985) and procedures as reported in Trontin *et al.* (2002). PPT at 0.1 mg l^{-1} was used as selective agent during the whole selection phase (Trontin *et al.* 2007, 2013). Briefly, ESMs (500 mg f.m.) were co-cultivated (1-2 weeks) with *A. tumefaciens* harbouring the OE-*PpMYB8* or RNAi-*PpMYB8* vector. After decontamination with appropriate antibiotics (1-2 weeks), ESMs

were cultivated for 13 weeks on selection medium. PPT-resistant embryogenic lines (PPT+) were collected and further proliferated on selection medium for 4 weeks and then on standard mLV proliferation medium for 4 to 6 weeks (Morel *et al.,* 2014). At this step a sample of PPT+ lines was cryopreserved (Harvengt, 2005) and PCR-screened to confirm their transgenic status. Experiments were designed to generate at the same time both transgenic and control lines transformed with empty binary vector (EV). Non-transgenic (NT) control lines were also obtained during the same experiments after cocultivation with the C58pMP90 *A. tumefaciens* strain deprived of any binary vector. Somatic embryo (SE) development from selected cryopreserved transgenic embryogenic lines was achieved on mLV-based maturation medium within 12 weeks following the method reported in Morel *et al.* (2014). Cotyledonary SE were harvested and stored in the dark at 4°C on maturation medium deprived of abscisic acid until germination assays. Cotyledonary SE from transgenic and control EV lines were germinated *in vitro* for ca. two weeks on a modified DCR medium. Germinated SE were then transplanted into plugs and grown for 4-8 additional weeks to obtain rooted plantlets of sufficient size and quality to be progressively acclimatized to soil conditions into the greenhouse. Plants obtained from embryogenic lines transformed with the corresponding empty vector (EV) and non-transgenic lines (NT) were used as controls.

Yeast manipulation and β-galactosidase assay

pMW3 constructs carrying the different deletions of the PpADT-A and PpADT-D regulatory regions were integrated into the genome of Saccharomyces cerevisiae YM4271 as described by Deplancke et al. (2006). Twelve yeast bait independent lines for each reporter construct were analyzed into Yeast Nitrogen Base with glucose (YNB) media plates lacking uracil, supplemented with X-Gal, to estimate the reporter self-activation level. After four days of incubation at 30º C, three bait lines with the lowest self-activation level were selected for each bait construct, and transformed with the prey construct pDEST22-PpMYB8, or the empty vector as control. Yeast transformations were plated into YNB-glucose media without uracil and Trp and incubated at 30º C until yeast colonies were grown. The presence of both the bait and prey constructs was confirmed by on-colony PCR prior to β-galactosidase experiment.

For the β-galactosidase assay with ONPG as substrate, two independent colonies for each bait-prey combination were grown overnight at 30º C and 200 rpm into YNB-glucose liquid medium lacking uracil and Trp. 0.5 mL of the saturated overnight cultures was used to inoculate 10 mL of Yeast Peptone Dextrose (YPD) liquid medium,

previously warmed to 30º C. YPD tubes were incubated during 4 hours at 30º C and 200 rpm and 1.5 mL was employed from each tube for the assay, as described by Clontech's Yeast Protocol Handbook (Various authors, 2009). Reactions were incubated from 45 to 90 min until yellow color was visible, and stopped with Na_2CO_3 1 M. Absorbance was measured at 420 nm.

References

Litvay JD, Verma DC, Johnson MA. 1985. Influence of loblolly pine (*Pinus taeda* L.) culture medium and its components on growth and somatic embryogenesis of the wild carrot (*Daucus carota* L.). Plant and Cell Reports **4**, 325-328.

Gupta PK, Durzan DJ. 1985. Shoot multiplication from mature trees of Douglas-fir (*Pseudotsuga menziesii*) and sugar pine (*Pinus lambertiana*). Plant and Cell Reports **4**, 177-179.

Deplancke B, Vermeirssen V, Arda HE, Martinez NJ, Walhout AJ. 2006. Gateway-compatible yeast one-hybrid screens. Cold Spring Harbour Protocols pii: pdb.prot4590.

Various authors. 2009. Clontech: Yeast Protocol Handbook. VI.D.: Liquid Culture Assay Using ONPG as Substrate, 27. Consulted online in the following link: www.clontech.com/xxclt_ibcGetAttachment.jsp?cItemId=17602
